# Long-chain fatty acids alter transcription of *Helicobacter pylori* virulence and regulatory genes

**DOI:** 10.7717/peerj.12270

**Published:** 2021-11-01

**Authors:** Hilda A. Valdez-Salazar, Miguel A. Ares, Francisco J. Fernández, J Antonio Ibarra, Javier Torres, Víctor H. Bustamante, Miguel A. De la Cruz

**Affiliations:** 1 Unidad de Investigación Médica en Enfermedades Infecciosas y Parasitarias, Instituto Mexicano del Seguro Social, Mexico City, Mexico; 2 Posgrado en Biología Experimental, DCBS., Universidad Autónoma Metropolitana (UAM) Iztapalapa, Mexico City, Mexico; 3 Departamento de Microbiología, Escuela Nacional de Ciencias Biológicas, Instituto Politécnico Nacional, Mexico City, Mexico; 4 Laboratorio de Ingeniería Genética y Metabolismo Secundario, Departamento de Biotecnología, Universidad Autónoma Metropolitana, Mexico City, Mexico; 5 Departamento de Microbiología Molecular, Instituto de Biotecnología, Universidad Nacional Autónoma de México, Cuernavaca, Morelos, Mexico

**Keywords:** LCFA, *Helicobacter pylori*, Virulence factors, Transcription

## Abstract

Infection with *Helicobacter pylori* is one of the most important risk factors for developing gastric cancer (GC). The type IV secretion system (T4SS) encoded in the *cag* pathogenicity island is the main virulence factor of *H. pylori* associated with GC. Additionally, other virulence factors have been shown to play a role in the *H. pylori* virulence, such as vacuolizing cytotoxin (VacA), urease, flagella, and adhesins. Long-chain fatty acids (LCFAs) are signaling molecules that affect the transcription of virulence genes in several pathogenic bacteria such as *Salmonella enterica*, *Vibrio cholerae*, *Pseudomonas aeruginosa* and *Mycobacterium tuberculosis*. However, the effect of LCFAs on the transcription of *H. pylori* virulence and regulatory genes remains unknown. Here we analyzed whether the transcription of virulence genes that encode T4SS and cellular envelope components, flagellins, adhesins, toxins, urease, as well as the transcription of different regulatory genes of the *H. pylori* strain 26695, are altered by the presence of five distinct LCFAs: palmitic, stearic, oleic, linoleic, and linolenic acids. Palmitic and oleic acids up-regulated the transcription of most of the virulence genes tested, including *cagL*, *cagM*, *flaB*, *sabA*, *mraY* and *vacA*, as well as that of the genes encoding the transcriptional regulators NikR, Fur, CheY, ArsR, FlgR, HspR, HsrA, Hup, and CrdR. In contrast, the other LCFAs differentially affected the transcription of the virulence and regulatory genes assessed. Our data show that LCFAs can act as signaling molecules that control the transcription of the *H. pylori* virulome.

## Introduction

*Helicobacter pylori* is a Gram-negative microaerophilic bacterium that colonizes the stomach mucosa during the entire life of many humans. *H. pylori* has a spiral shape and measures around 3 and 0.5 micrometers in length and diameter, respectively. It possesses multiple flagella (4–6) at one of its ends, which mediate motility and colonization of the gastric epithelium; these flagella are composed of proteins called flagellins (50–60 kDa) that are encoded by the *flaA* and *flaB* genes (Cover et al., 1991; Eaton, Morgan & Krakowka, 1992; Josenhans, Labigne & Suerbaum, 1995). Its most outstanding biochemical characteristic is the abundant production of the urease enzyme, which catalyzes the hydrolysis of urea into ammonia and carbon dioxide, neutralizing the stomach acidic pH (Dunn et al., 1990). Other virulence genes described in *H. pylori* for gastric colonization and tissue damage include those encoding a Type IV secretion system (T4SS), VacA vacuolizing cytotoxin, and adhesins (Molnar et al., 2010). The *H. pylori* T4SS is encoded by genes located in the 40 kb *cag* pathogenicity island (Tegtmeyer, Wessler & Backert, 2011). It is a complex of proteins forming a needle that allows the secretion and translocation of effector molecules such as the CagA protein within gastric epithelial cells (Yuan, Wang & Wang, 2018). VacA is a cytotoxin that forms pores in the membranes of the epithelial cells and allows the exit of anions and urea; it also favors the separation of epithelial junctions, which makes possible for nutrients to pass through the mucosal barrier to benefit the bacteria (Palframan, Kwok & Gabriel, 2012; Rassow & Meinecke, 2012). In addition to mediate adhesion and colonization of host gastric mucosa by *H. pylori*, the adhesins enhance the translocation of CagA into the host cells and therefore the release of the proinflammatory cytotoxin IL-8 (Bonsor & Sundberg, 2019). BabA (Blood group Antigen-Binding Adhesin) is an adhesin with characteristics similar to those observed in the Lewis blood group (Ilver et al., 1998; Yamaoka, 2008). SabA (Sialic acid-binding adhesin) is another adhesin that mediates a strong association between the bacterium and the epithelium cells, and thus facilitates the action of other virulence factors, resulting in an increase in the inflammation and damage to the gastric mucosa (Mahdavi et al., 2002). HopQ was recently described as an adhesin that specifically binds to human CEACAMs (carcino embryonic antigen-related cell adhesion molecules) (Javaheri et al., 2016; Koniger et al., 2016). Other cell envelope components such as peptidoglycan and lipopolysaccharide have been reported to be relevant in the physiology and virulence of *H. pylori* (Li et al., 2017; Sycuro et al., 2012). Penicillin-binding proteins (PBPs) are acyl-serine transferases that are involved in the assembly, integrity and dynamic of the peptidoglycan matrix (Sauvage et al., 2008). Although five PBPs have been identified in *H. pylori* 26695, PBP1 was the first identified and is currently the most studied PBP (Paul et al., 2001; Rimbara et al., 2008). Moreover, MraY is an integral membrane protein involved in cell wall biosynthesis with phospho-N-acetyl-muramyl-pentapeptide transferase activity, catalyzing the first of a sequence of lipid-linked steps that ultimately assemble the peptidoglycan layer of the bacterial cell wall (Krzyzek & Gosciniak, 2018). On another hand, WaaL ligase (lipid A-core-O-antigen-ligase) participates in the last step of lipopolysaccharide (LPS) biosynthesis, facilitating the transferring of the O polysaccharide from undecaprenyl pyrophosphate onto the lipid A-core (Hug et al., 2010).

Expression of *H. pylori* virulence factors is mainly controlled by 13 transcriptional regulators and three sigma factors (Danielli, Amore & Scarlato, 2010; De la Cruz et al., 2017). Lipids are crucial biomolecules that function as structural components and as energy sources. Long-chain fatty acids (LCFAs) are lipids that, in addition to these functions, act as signaling molecules in both eukaryotic and prokaryotic cells. In this sense, the pathogenic bacteria have developed signal transduction mechanisms able to sense LCFAs to control the expression of their virulence genes. In *Salmonella enterica* serotype Typhimurium, oleic, myristic and palmitic acids repressed the expression of the genes located in the pathogenicity island 1 (SPI-1), which encodes a Type III secretion system (T3SS) that is necessary for the invasion of intestinal epithelial cells (Golubeva et al., 2016). Arachidonic, oleic and linoleic acids suppress the production of cholera toxin of *Vibrio cholerae* (Chatterjee, Dutta & Chowdhury, 2007). LCFAs are important for the replication of *Pseudomonas aeruginosa* in a murine model of lung infection (Kang et al., 2010). In the case of *Mycobacterium tuberculosis*, the LCFAs affect the expression of some virulence genes, specifically those related with the granuloma formation (Ehrt & Schnappinger, 2007; Rodriguez et al., 2014). In addition to palmitic (16:0), stearic (18:0), oleic (18:1), and linoleic (18:2) acids, we analyzed the role of linolenic (18:3) acid, which is an essential fatty acid for humans (together to linoleic acid) and presents three double C-C bonds. Nevertheless, the effect of these LCFAs on the *H. pylori* virulence and/or regulatory genes has not been reported.

In this study, the transcription of *H. pylori* virulence genes encoding T4SS components, adhesins, urease, flagella, peptidoglycan, and LPS, as well as that of several regulatory genes, was quantified in the presence of palmitic, stearic, oleic, linoleic, and linolenic acids. Our results showed that both palmitic and oleic acids increase the transcription of most virulence genes tested, which seems to be an effect of the upregulation of different transcription factors by these LCFAs*.* In contrast, linoleic acid diminished the transcription of some virulence genes such as *flaB*, *cagL, ureB* and *babA*. Our work demonstrated that LCFAs can act as signaling molecules that control the expression of *H. pylori* virulence factors.

### Materials and methods

### Bacterial strains and culture conditions

*H. pylori* strain 26695 was grown as was previously described (De la Cruz et al., 2017). Cultures were incubated for 1 h in the presence or not of individual LCFAs at a final concentration of 250 µM. 0.05% tyloxapol was added as surfactant. Fold-changes in transcription was determined by calculating the relative expression of virulence genes in the presence or not of the distinct LCFAs. On three different days, assays were performed in triplicate and the data shown are the mean of the results obtained.

### RNA isolation and quantitative RT-PCR

Extraction of total RNA was performed as previously described (Jahn, Charkowski & Willis, 2008; De la Cruz et al., 2017). cDNA was synthesized using 1 µg of RNA, 0.2 µg/µL of random hexamer primers and 2 U/µL of M-MulV-RT (Reverse transcriptase of Moloney Murine Leukemia Virus; Thermo Scientific). Quantitative real-time PCR was performed in a LightCycler 480 instrument (Roche) to quantify the gene expression levels. For LightCycler reactions, a master mix of the following components was prepared: 3.0 µL of PCR-grade water, 1.0 µL (10 µM) of forward primer, 1.0 µL (10 µM) of reverse primer, 10 µL of 2x Master Mix and 5.0 µL of cDNA (50–100 ng). A multiwell plate containing all samples was loaded into the LightCycler 480 instrument. Amplification was performed in triplicate wells for each sample analyzed from three independent experiments. In each set of reactions, 16S rRNA (*rrnA16S*) was used as a reference gene for normalization of cDNA amount. Real-time PCR analysis was performed using the following optimized assay conditions: (1) denaturation program (95 °C for 10 min); amplification and quantification program repeated for 45 cycles (95 °C for 10 s, 58 °C/59 °C (depending Tm; see Table 1) for 20 s, 72 °C for 30 s with a single fluorescence measurement); (2) melting curve program (95 °C for 10 s, 65 °C for 1 min with continuous fluorescence measurement at 97 °C) and finally; (3) a cooling step at 40 °C for 10 s. Contamination with DNA was verified in all reactions by running an RT-minus (after 45 qPCR cycles). 2^−^^ΔΔ^^Ct^ method was used to calculate the relative gene expression (Livak & Schmittgen, 2001).

**Table 1:** Oligonucleotides for qRT-PCR used in this study.

Gene	Sequence 5′–3′	*Target gene*	*Tm* (°*C)*
*cagA*	AGC AAA AAG CGA CCT TGA AA AGC CAA TTG CTC CTT TGA GA	*hp0547*	*58*
*cagL*	AGA CCA ACC AAC AAG TGC TC ATC TCC TCT CAA AGC AAT GGC	*hp0539*	*58*
*cagM*	TAC CAA CAA GCC GCA TAC AA ACA AAA CTC CAA ACG CAA CC	*hp0537*	*58*
*flaA*	ACA AGT GGA TGG CGT GAA TG ACG CTC GCA TAG GCT TTA AC	*hp0601*	*59*
*flaB*	AAA CCA AAG CCG TTC AAG CC TTG GCC GTT AAA GCT TGT GG	*hp0115*	*58*
*ureA*	AGC CTG GCG AAG AAA AAT CC TGC TTT CGT TGT CTG CTT GC	*hp0073*	*59*
*ureB*	ATC AAA GGC GGG TTC ATT GC AGC CGC TTG AGA CAC AAA AG	*hp0072*	*58*
*vacA*	AAG CAC CAT TTG CCT TTG AC CGT TCA ATT TCA GCG TGC TA	*hp0887*	*58*
*sabA*	TGG CGT TTC AAA CAC ATC CG ACC GCT TGT TGC AAA ATG GG	*hp0725*	*58*
*babA*	ACA CCA TCA ACG AAG CAT GC AAA GCG CCG CAT ATT TTC CC	*hp1243*	*58*
*hopQ*	AAG CGC AAA ACC TAG CCA AC ATT CAA GGC GCC GTT ATT CG	*hp1177*	*59*
*mraY*	CCA TTT CTA GCT TCG TGC CAA G ACA ACA CGC TCG CAA CAA TG	*hp0493*	*59*
*pbp1*	CCC CAG CAA TTA TTC TCG CAA G TTC AAA GCC AAG CTG ATC GC	*hp0597*	*59*
*waaL*	GCT TGC TTT TTG CCA GTT GC TTG CAC CAA TGA GAG CAA GC	*hp1039*	*59*
*nikR*	CAT CCG CTT TTC GGT TTC CAT GTC GCG CAC TAA TTC TG	*hp1338*	*58*
*fur*	GAA GAA GTG GTG AGC GTT TTG CCT TTT GGC GGA TAG AAT GC	*hp1027*	*58*
*cheY*	TGG AAG CTT GGG AGA AAC TG CAG AGC GCA CCT TTT TAA CG	*hp1067*	*58*
*hrcA*	TTT CTT GCG CAC TGG GTT AC GAA AGA AGC AGC GAT TGA GC	*hp0111*	*58*
*arsR*	GAG CGA GTT TTT GCT CCA AC GCC CGT CTA AAT TAG GCA AAG	*hp0166*	*58*
*flgR*	CAG GCC TTA AAA GTC GCA AG CGC TAT AAA AGG GTG CTT GG	*hp0703*	*58*
*hspR*	CGG GCG TGG ATA TTA TCT TG TGT TTG TGC AGA GCG TCT TG	*hp1025*	*58*
*hsrA*	GGA AGA AGT CCA TGC GTT TG CAA ACG AGC CTC AAT CCT TG	*hp1043*	*58*
*hup*	GTG GAG TTG ATC GGT TTT GG TTA GGC ACC CGT TTG TCT TC	*hp0835*	*58*
*crdR*	CTT AGG CGT GGC TAA AAT GC CAA ACG CCC CAA AAA CAC	*hp1365*	*58*
*16S*	GTG TGG GAG AGG TAG GTG GA GTT TAG GGC GTG GAC TAC CA	*rrnA16S*	*58*

### Quantification of IL-8 secretion

Quantification of IL-8 was performed as previously described (Vazquez-Jimenez et al., 2016). AGS gastric epithelial cells were infected at a multiplicity of infection (MOI) of 100 with *H. pylori* grown at exponential phase in BB + 10% FBS (OD_600nm_ = 0.8) under the presence of individual LCFAs (250 µM). After 6 h of infection at 37 °C under microaerophilic conditions, the culture supernatant was collected and stored at −80 °C until tested. IL-8 concentration was quantified in each supernatant using a commercial enzyme-linked immunosorbent assay for human IL-8 (BD Biosciences, San Diego, CA, USA), following the manufacturer’s instructions. The supernatant of uninfected AGS cells was tested as negative control. Quantifications of IL-8 were performed in triplicate on three different days

### Soft agar motility assay

Soft agar plates composed of 0.35% Bacto agar, 1.0% tryptone (BD), 2.5% decomplemented fetal bovine serum were prepared as described previously (Sanders, Andermann & Ottemann, 2013). 10 µL of a *H. pylori* bacterial suspension (OD_600nm_ = 0.8) in BB + 10% FBS was inoculated onto the soft agar supplemented with individual LCFAs (250 µM) and the plates were incubated at 37 °C under microaerophilic conditions. Migration diameter was measured after three days. On three different days, swimming assays were performed in triplicate.

### Cell adherence assays

Cultured AGS gastric epithelial cells were used in adherence assays as described previously (De la Cruz et al., 2017). As inocula, cultures of *H. pylori* at exponential phase in BB + 10% FBS (OD_600nm_ = 0.8) under the presence of individual LCFAs (250 µM) were used. The cells were infected at a multiplicity of infection (MOI) of 100 for 6 h at 37 °C under microaerophilic conditions, washed thrice with PBS to remove unbound bacteria, and subsequently treated with 1 mL of 0.1% TritonX-100 for 15 min. Following the lysis of AGS cells, CFUs were quantified by plating out 10-fold dilutions of the bacterial suspensions. On three different days, quantifications were performed in triplicate, and the mean results were expressed as adhering CFUs/mL.

### Biofilm formation

Biofilm formation on abiotic surface (polystyrene) was analyzed using 96-well polystyrene plates as described previously (Hathroubi, Zerebinski & Ottemann, 2018). *H. pylori* cultures were diluted to an OD_600_ of 0.15 in *Brucella* broth supplemented with 2% decomplemented fetal bovine serum in the presence of individual LCFA (250 µM). The plates were incubated for three days at 37 °C under microaerophilic conditions. After incubation, the supernatant was discarded and the wells were rinsed thrice with PBS. The biofilm was stained with 0.1% Crystal Violet (Merck). After washing with PBS, the adsorbed dye was recovered with 70% ethanol and quantified at an OD_590_ with a spectrophotometer (Multiskan Ascent; Thermo Scientific). On three different days, quantifications of the biofilm formation were performed in triplicate.

### Heatmap construction

Heatmapper web server (http://www.heatmapper.ca/expression/) was used to illustrate the gene expression changes between the untreated control and different LCFA treatment groups.

### Statistical analysis

For statistical differences, one-way ANOVA followed by the Tukey’s comparison test was performed using GraphPad Prism (version 6.0c for Mac OS X), La Jolla, California, USA. *p* < 0.05 was considered statistically significant.

## Results

### Effect of LCFAs on the expression of genes encoding T4SS components

**Figure 1:**
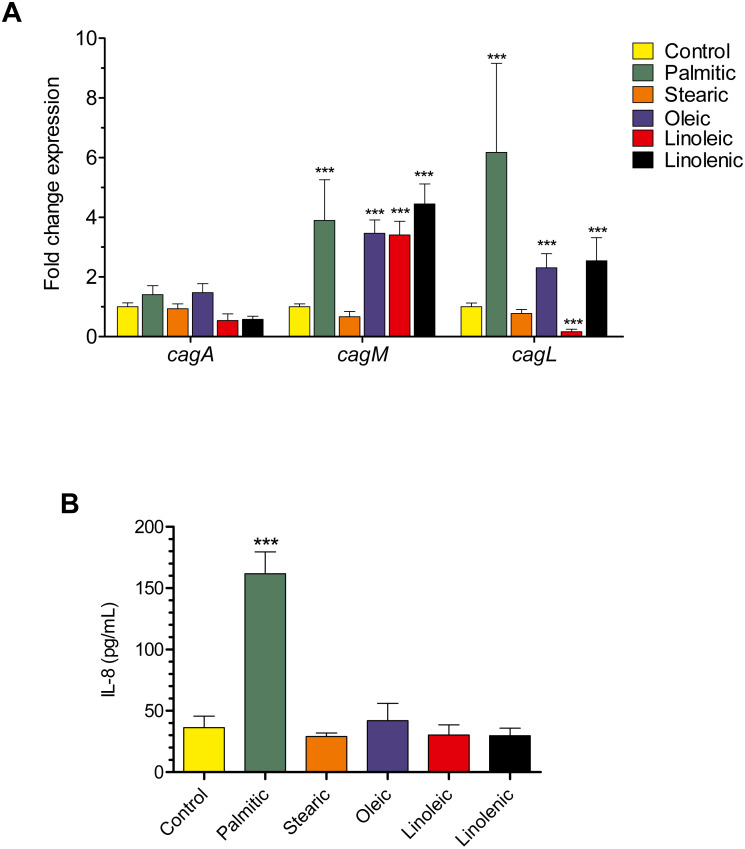
Effect of the palmitic, stearic, oleic, linoleic and linolenic acids on *cag*PAI expression. (A) Expression detected by RT-qPCR of the cag genes of *H. pylori* in logarithmic phase of growth in the presence or not of LCFA at a final concentration of 250 µM. *cagM* gene was up-expressed with palmitic (4-fold), oleic (3-fold), linoleic (3-fold) and linolenic acid (4-fold). *cagL* gene was up-regulated with palmitic (6-fold), and more discreetly with oleic (2-fold) and linolenic acid (2.5-fold); this gene was repressed with linoleic acid (6-fold). No statistically significant changes in *cagA* expression levels were observed. (B) ELISA analysis of IL-8 protein expression in supernatants of *H. pylori*-infected AGS cells at 6 h post-infection. Data represent the mean with standard deviations obtained from three separate experiments performed in triplicate. Statistically significant with respect to control: ***, *p* < 0.001.

To determine the effect of LCFAs on the expression of the *H. pylori* T4SS, the transcription of the *cagA, cagL*, and *cagM* genes was tested by RT-qPCR in the presence of the palmitic, stearic, oleic, linoleic, and linolenic acids. The transcription of both *cagL* and *cagM* genes, which encode the pilus structure and a transmembrane protein of the T4SS, respectively, significantly induced in the presence of palmitic, oleic and linolenic acids (Fig. 1A). Interestingly, linoleic acid exerted a positive and negative effect on the transcription of *cagM* and *cagL*, respectively (Fig. 1A). In contrast, the transcription of the *cagA* gene, which encodes the oncogenic cytotoxin translocated into host cells through the T4SS, was not significantly affected by any of the LCFA tested (Fig. 1A). Interestingly, the secretion of IL-8 from AGS cells, which requires the T4SS, was significantly boosted only in response to *H. pylori* grown in the presence of palmitic acid, but not the other LCFAs tested (Fig. 1B). Our data showed that palmitic, oleic and linolenic acids enhance the transcription of T4SS structural components of *H. pylori*. Intriguingly, we found that only the palmitic acid induces the IL-8 secretion from gastric epithelial cells mediated by *H. pylori*.

### Effect of LCFAs on the expression of genes encoding flagellins and adhesins

**Figure 2:**
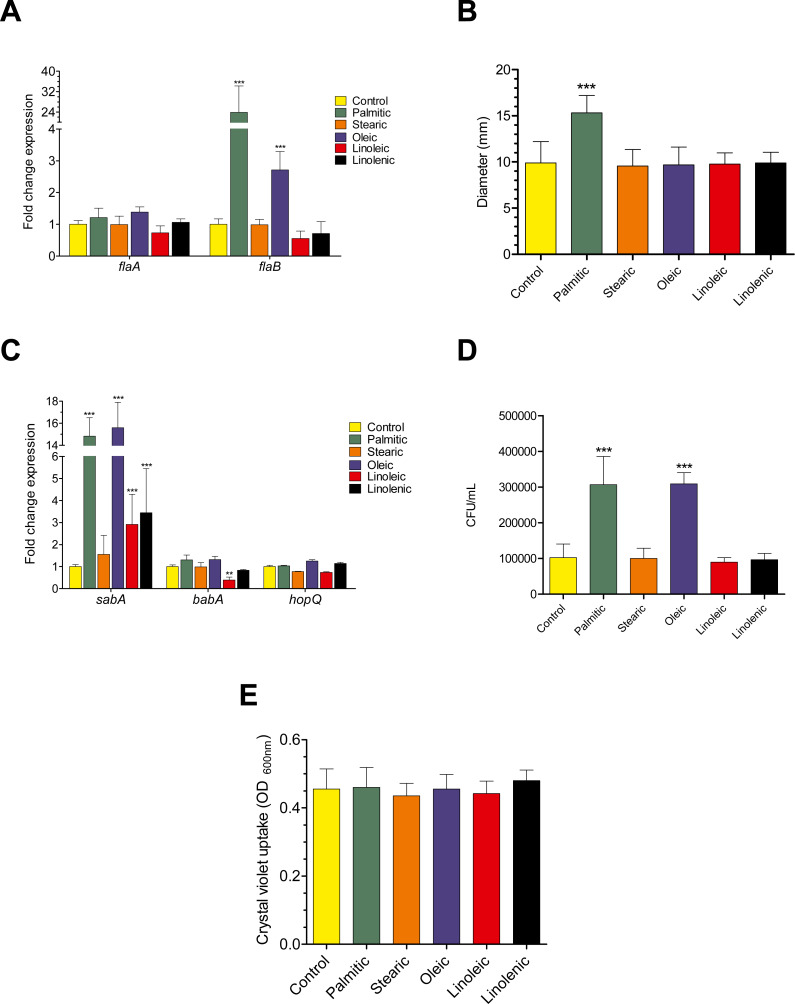
Effect of LCFAs on the expression of flagellar and adhesin genes, as well as on the motility and adherence of *H. pylori*. (A) Expression measured by RT-qPCR of the *flaA* and *flaB* flagellar genes in logarithmic phase of growth. *flaB* gene was highly expressed in the presence of palmitic (24-fold) and oleic (3-fold) acids. (B) Effect of LCFAs on the *H. pylori* motility on soft-agar plates, which is expressed as the diameter of migration distances of the colonies. (C) Expression detected by RT-qPCR of the *sabA, babA*, and *hopQ* adhesins genes in logarithmic phase of growth. *sabA* gene was up-regulated in the presence of the palmitic (15-fold), oleic (16-fold), linoleic (3-fold), and linolenic (3-fold) acids. babA gene was also down-regulated in the presence of linoleic acid (2.5-fold). (D) Levels of H. pylori adherence to AGS cells. Bacteria were grown in the presence of each LCFA tested and then inoculated to an AGS cell monolayer at a MOI of 100 for 6 h at 37 °C in a humidified atmosphere with 5% CO2. (E) Quantification of biofilm formation in the presence or not of LCFAs to a final concentration of 250 µM was determined by measuring Crystal Violet uptake by bacteria. All these experiments were performed in the presence or not of a final LCFAs concentration of 250 µM. Data expressed as fold-change expression, CFU/mL or absorbance, represent the mean with standard deviations obtained from three separate experiments performed in triplicate. Statistically significant with respect to control: **, *p* < 0.01; ***, *p* < 0.001.

To determine whether LCFAs affect the motility and adherence of *H. pylori*, we analyzed the transcription of flagellar and adhesins genes in the presence of the palmitic, stearic, oleic, linoleic, and linolenic acids. Both *flaA* and *flaB* flagellar genes*,* which encode the major and minor flagellin, respectively, as well as the *sabA*, *babA* and *hopQ* adhesin genes were assessed. The transcription of *flaB* was increased 24 and 3-fold in the presence of palmitic and oleic acids, respectively, and it was not affected by the other LCFAs tested; in contrast, none of the LCFAs assessed modified the transcription of *flaA* (Fig. 2A). In consistent with its highly inductive effect on *flaB* gene expression, the palmitic acid also significantly (*p* < 0.001) enhanced the *H. pylori* motility on soft agar (Fig. 2B). On another hand, the transcription of *sabA* was significantly increased in the presence of most LCFAs tested; palmitic and oleic acids induced up to 15-fold the transcription level of this gene (Fig. 2C). In agreement with these expression data, both palmitic and oleic acids increased 3-fold the adherence capacity of *H. pylori* to AGS cells (Fig. 2D); LCFAs evaluated did not affect the transcription of both *babA* and *hopQ* genes, with the exception of linoleic acid that reduced 2.5-fold the transcription level of *babA* (Fig. 2C). In addition, none of the LCFA tested affected the biofilm formation by *H. pylori* (Fig. 2E), showing that palmitic and oleic acids did not affect the adherence of *H. pylori* to an abiotic surface. Hence, our results support that palmitic and oleic acids enhance the motility and adherence of *H. pylori* by upregulating the transcription of the *flaB* and *sabA* genes, respectively.

### Palmitic and oleic acids stimulate the transcription of the *mraY* gene for peptidoglycan synthesis

**Figure 3:**
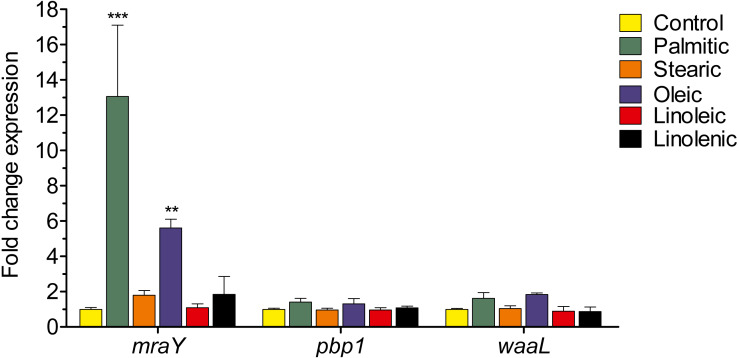
Effect of LCFAs on the expression of cell envelope genes. Expression quantified by RT-qPCR of the *H. pylori* envelope genes in logarithmic phase of growth in the presence or not of LCFAs at a final concentration of 250 µM. mraY gene was highly up-regulated with palmitic (13-fold) and oleic (6-fold) acids. No statistically significant changes in expression levels for *pbp1* and *waaL* were observed. Data represent the mean with standard deviations obtained from three separate experiments performed in triplicate. Statistically significant with respect to control: **, *p* < 0.01; ***, *p* < 0.001.

In order to determine the effect of LCFAs on the expression of *H. pylori* cell envelope components, we quantified the transcription level of *mraY*, *pbp1*, and *waaL* genes, which encode phospho-N-acetylmuramoyl-pentapeptide-transferase, penicillin-binding protein 1A, and lipid A core-O-antigen ligase, respectively. The transcription level of *mraY* gene was increased 13- and 6-fold in the presence of the palmitic and oleic acids, respectively, whereas it was not affected by the other LCFAs tested (Fig. 3). In contrast, none of the LCFAs evaluated significantly affected the transcription of *pbp1* and *waaL* (Fig. 3). Thus, these results indicate that the palmitic and oleic acids increase the transcription level of the *mraY* gene required for the peptidoglycan synthesis.

### Effect of LCFAs on the transcription of the *vacA* and *ureAB* genes

**Figure 4:**
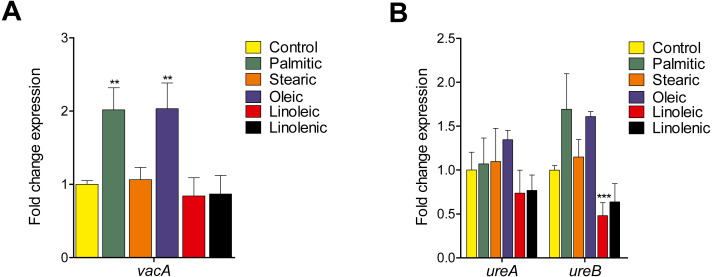
Effect of LCFAs on the expression of the *vacA* and *ureAB* genes. Expression determined by RT-qPCR of the *vacA* gene encoding the vacuolizing cytotoxin (A), and the *ureA* and *ureB* genes that encode the urease enzyme (B), in logarithmic phase of growth in the presence or not of LCFAs at a final concentration of 250 µM. Both palmitic and oleic acids enhanced the *vacA* transcription around 2-fold. The transcription level of ureA was unaffected by the LCFAs tested and that of ureB was expressed 2-fold in the presence of linoleic acid. Data represent the mean with standard deviations obtained from three separate experiments performed in triplicate. Statistically significant with respect to control: **, *p* < 0.01; ***, *p* < 0.001.

In *H. pylori*, VacA cytotoxin and urease enzyme are required for the eukaryotic cell vacuolization and bacterial survival under acidic pH, respectively (Mobley, 1996; Palframan, Kwok & Gabriel, 2012). To determine whether LCFAs affect these virulence phenotypes of *H. pylori*, we quantified the transcription level of the *vacA*, *ureA*, and *ureB* genes. The transcription of *vacA* was significantly boosted by two-fold (*p* <  0.01) in the presence of palmitic and oleic acids but not affected by the other LCFAs tested (Fig. 4A). While the transcription level of *ureA* was unaffected by any of the LCFAs tested, a significant two-fold down-regulated gene expression was observed in *ureB* when *H. pylori* was treated with linoleic acid (Fig. 4B).

### LCFAs differentially regulate the transcription of *H. pylori* regulatory genes

**Figure 5:**
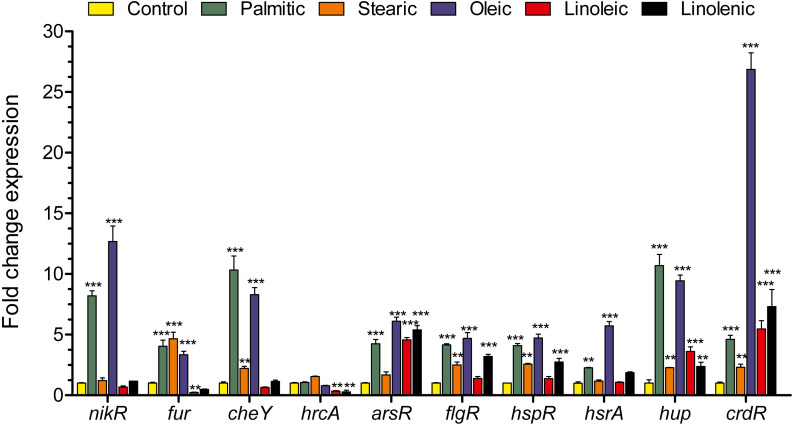
Effect of LCFAs on the expression of different regulatory genes. Expression detected by RT-qPCR of the *nikR, fur, cheY, hrcA, arsR, flgR, hspR, hsrA, hup*, and *crdR* regulatory genes in logarithmic phase of growth in the presence or not of LCFAs at a final concentration of 250 µM. Both palmitic and oleic acids increased the transcription levels of *nikR, fur, cheY, arsR, flgR, hspR, hsrA, hup*, and *crdR*. Both linoleic and linolenic acids up- or down-regulated the transcription levels of several of these genes such as *fur, hrcA, arsR, flgR, hspR, hup*, and *crdR*. Data represent the mean with standard deviations obtained from three separate experiments performed in triplicate. Statistically significant with respect to control: **, *p* < 0.014; ***, *p* < 0.001.

*H. pylori* genome encodes a limited number of transcriptional regulators, which control the expression of genes involved in bacterial survival, metabolism and pathogenicity. We determined the effect of the palmitic, stearic, oleic, linoleic, and linolenic acids on the transcription of genes encoding several of these transcriptional factors. With the exception of *hrcA*, the rest of the regulatory genes assessed (*nikR*, *fur*, *cheY*, *arsR*, *flgR*, *hspR*, *hsrA*, *hup* and *crdR*) showed increased transcription levels in the presence of the palmitic and oleic acids, while the linoleic and linolenic acids up or down-regulated the transcription of several of these genes such as *fur*, *hrcA*, *arsR*, *flgR*, *hspR*, *hup*, and *crdR* (Fig. 5). Therefore, these results reveal that the LCFAs tested exert a differential regulatory effect on genes encoding several transcriptional regulators of *H. pylori*.

## Discussion

**Figure 6:**
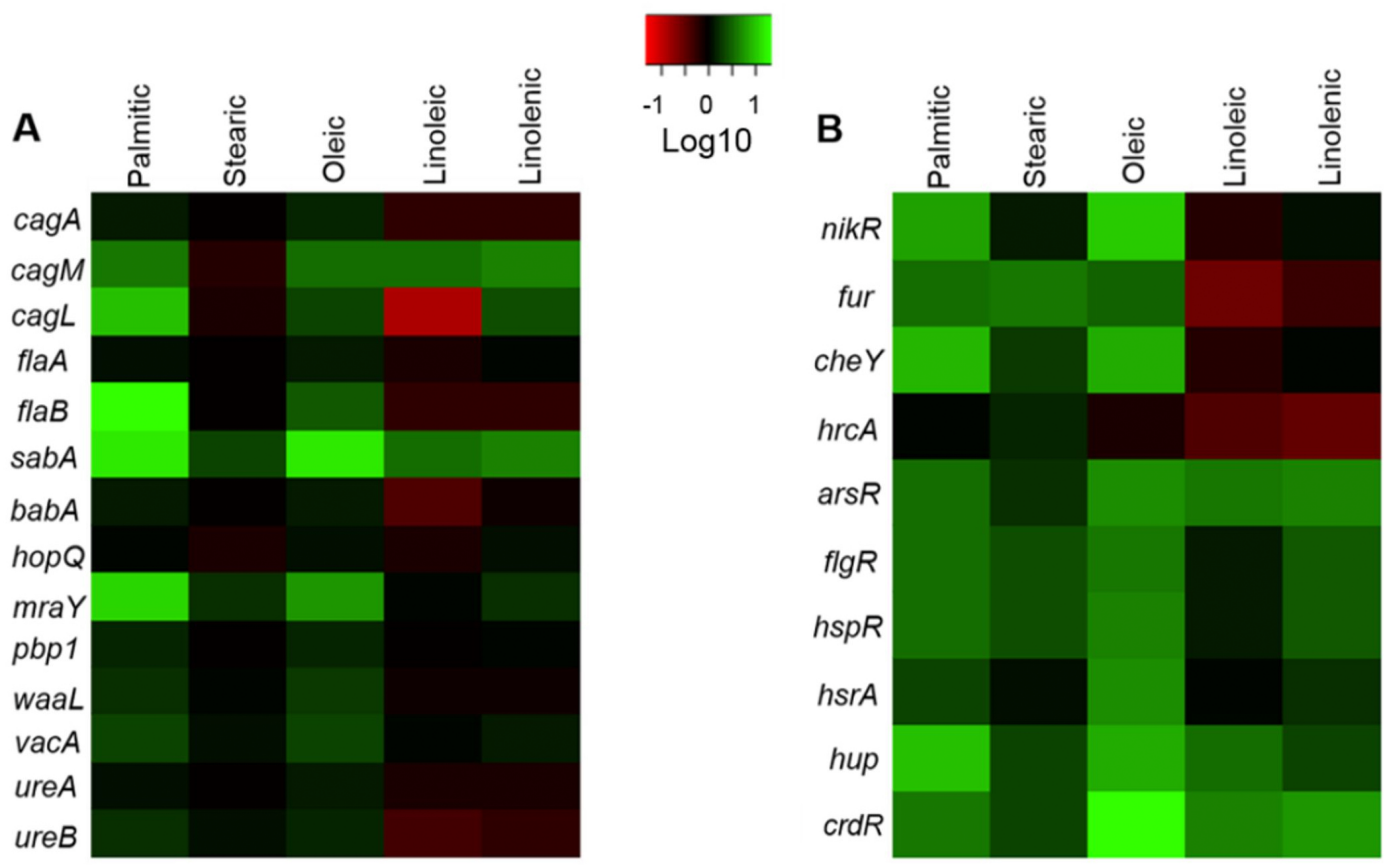
Summary of the effects of LCFAs on the expression of virulence and regulatory genes of *H. pylori*. Heatmap of the relative transcriptional expression of virulence (A) and regulatory (B) genes in the presence of LCFAs: palmitic, stearic, oleic, linoleic and linolenic. Relative gene expression values are shown as fold-changes in a Log10 scale. The color-coding scale denotes up-regulation in green and down-regulation in red.

*H. pylori* is highly adapted to one specific niche: the human gastric mucosa. Our group has described that environmental cues such as acidic pH, iron, nickel or copper are crucial to control the transcription of *H. pylori* virulence genes (Cardenas-Mondragon et al., 2016; De la Cruz et al., 2017). In this context, some reports indicate that LCFAs can act as signaling molecules, altering the transcription of genes that encode virulence factors in different pathogenic bacteria (Chatterjee, Dutta & Chowdhury, 2007; Ehrt & Schnappinger, 2007; Golubeva et al., 2016; Kang et al., 2010). LCFAs are mainly found in both prokaryotic and eukaryotic membrane phospholipids, LPS lipid A, bile salts and the daily diet. Palmitic, stearic, oleic, linoleic, and arachidonic acids are the main LCFAs that form the eukaryotic membrane phospholipids, which can be synthesized *de novo* or modified by deacylation/reacylation reactions (Hulbert, Kelly & Abbott, 2014; Schmid, Deli & Schmid, 1995). In this work we evaluated the effect of the palmitic, stearic, oleic, linoleic, linolenic acids on the transcription of genes encoding major virulence factors and different transcriptional regulators of *H. pylori*. The palmitic and oleic acids were the two main LCFAs that mostly up-regulated the virulence genes assessed, such as *flaB, sabA, cagM, cagL, vacA* and *mraY* (Fig. 6A), which are involved in motility, adherence, and cell wall synthesis*.* The palmitic and oleic acids also stimulated the transcription of almost all the genes encoding transcriptional factors tested (Fig. 6B). On another hand, the linoleic and linolenic acids similarly affected two clusters of regulatory genes: *nikR*, *fur*, *cheY*, and *hrcA* were down-regulated, whereas *arsR*, *flgR*, *hspR*, *hsrA*, *hup*, and *crdR* were up-regulated by these LCFAs.

Some LCFAs have been shown to affect the function of transcriptional factors of different families. HilD and ToxT are two AraC-type transcription factors that act as master regulators of virulence genes in *Salmonella* and *Vibrio*, respectively (Ellermeier & Slauch, 2007; Weber & Klose, 2011). The palmitic and oleic acids repress the expression of *hilD* and *toxT*; moreover, the oleic acid directly binds to and thus block the DNA-binding activity of HilD and ToxT (Cruite et al., 2019; Golubeva et al., 2016). Besides, the palmitic and oleic acids reduce the DNA-binding affinity of TetR-type regulators Rv3249c/Rv1816 and TfmR of *M. tuberculosis* and *Xanthomonas citri,* respectively (Delmar et al., 2015; Teper, Zhang & Wang, 2019). Therefore, it is reasonable to propose that the palmitic and oleic acids also interact with some positive or negative transcriptional regulators of *H. pylori* and thus affect the expression of virulence genes in this bacterium.

With respect to the effect of LCFAs on the expression of *cag*PAI, the palmitic, oleic and linolenic acids activated the transcription of the *cagL* and *cagM* genes, which encode the T4SS structural components, whilst the transcription of the *cagA* cytotoxin gene was not significantly affected by any of the LCFAs tested. In addition, IL-8 secretion from AGS cells was stimulated when *H. pylori* grew in the presence of the palmitic acid. Our results suggest that the palmitic acid could be a signaling molecule involved in CagA-independent stimulation of gastric mucosa inflammation. In agreement with this notion, it has been shown that CagL is able to stimulate the secretion of IL-8 independently of CagA translocation (Gorrell et al., 2013). This CagA-independent inflammation could be supported by the low association between dyspepsia and CagA+ *H. pylori* strains observed in some worldwide patient cohorts (Andreolla et al., 2016; Bommelaer et al., 2001; Heikkinen et al., 1998; Nordenstedt et al., 2007; Vasiliou et al., 2014). The positive effects of the palmitic acid on the *sabA* transcription and on the adherence of *H. pylori* to AGS cells would enhance the inflammatory process, since SabA is an adhesin required for a strong interaction between the bacterium and gastric epithelial cells, which promotes the inflammation of the gastric mucosa (Mahdavi et al., 2002).

Although flagella and peptidoglycan have been reported as structures implicated in the biofilm formation by *H. pylori* (Hathroubi, Zerebinski & Ottemann, 2018), the presence of the palmitic acid, which up-regulates the transcription of *flaB* and *mraY* genes, encoding the FlaB flagellin and MraY enzyme related to peptidoglycan biosynthesis, respectively, did not affected the biofilm formation by *H. pylori*. These observations suggest that multiple factors are involved in the biofilm formation by *H. pylori* in response to different environmental cues.

Contrary to palmitic and oleic acids, the linoleic acid down-regulated the transcription of virulence genes such as *flaB, ureB, babA*, and *cagL,* and also that of the *fur* and *hrcA* regulatory genes. In *V. cholerae*, the oleic, linoleic and arachidonic acids repressed the expression of the *ctxAB* and *tcpA* genes, which encode cholera toxin and TCP type IV pilus, respectively (Chatterjee, Dutta & Chowdhury, 2007). Interestingly, the linoleic acid also increased the motility of *V. cholerae*, stimulating the expression of *flrA* gene, which encodes the top transcriptional regulator that controls the motility in this bacterium (Chatterjee, Dutta & Chowdhury, 2007). This dual effect on virulence or regulatory genes was also observed for some LCFAs in our study. For instance, the linolenic acid enhanced the transcription of the *sabA, arsR, hsrA, hup* and *crdR* regulatory genes, but decreased the transcription of the *hrcA* and *fur* regulatory genes.

Our data support regulatory pathways previously reported for some of the genes and phenotypes that we assessed in this study. For instance, the FlgR transcriptional regulator has been shown to induce the expression of *flaB* but not that of *flaA*, the genes encoding the flagellins of *H. pylori*, which are expressed as independent transcriptional units, and FlgR was demonstrated to be required for *H. pylori* motility (Spohn & Scarlato, 1999). Consistently with these reported findings, our results indicated that the palmitic and oleic acids up-regulated the transcription of both *flgR* and *flaB* genes, but not that of the *flaA* gene. Although stearic and linolenic acids induced the expression of *flgR* gene, these LCFAs did not affect the *flaB* expression suggesting others regulatory mechanisms. Furthermore, we found that the palmitic acid enhanced the *H. pylori* motility. As described above, the positive effect of a LCFA on the motility was observed for *V. cholerae*, since the linoleic acid increased the bacterial swimming by up-regulation of FlrA flagellar regulator (Chatterjee, Dutta & Chowdhury, 2007). Thus, our results suggest that mainly the palmitic acid acts as a signaling molecule that favors the *H. pylori* motility by inducing the expression of the *flaB* gene through the FlgR regulator. In this way, the transcription of *fur* and *ureB* genes were repressed in the presence of linoleic acid, supporting the observations that *ureB* is negatively controlled by 5′*ureB*-sRNA, which is repressed by ArsRS two-component system (Wen et al., 2011), and this bicisctronic gene is repressed by Fur regulator (Roncarati et al., 2016). In terms of *sabA*, it was previously reported that this adhesin gene was repressed by the ArsRS two-component system in the *H. pylori* strain J99 (Goodwin et al., 2008). However, our data showed that all LCFAs, except stearic acid, were able to stimulate the expression of both *arsR* and *sabA* genes, suggesting that the ArsRS two-component system positively regulates the *sabA* transcription in the *H. pylori* strain 26695 in the presence of these LCFAs. These differences in the *sabA* regulation between both strains could be associated with the poly-CT tract length within the 5′ portion of the coding region of each strain (Goodwin et al., 2008; Åberg et al., 2014), where ArsR DNA-binding activity could be modified by the local DNA structure and/or direct interaction with any LCFA.

Our study shows by the first time that LCFAs affect the expression of *H. pylori* virulence and regulatory genes. The elucidation of the mechanisms by which LCFAs alter the expression of these genes could help to develop anti-infective strategies to counteract the infections by *H. pylori*.

## Conclusions

The palmitic and oleic acids were the two main LCFAs that increased the transcription of virulence genes involved in motility, adherence, and cell wall synthesis of *H. pylori*. These same fatty acids enhanced the expression of almost all the regulatory genes tested. While the linoleic acid repressed the transcription of virulence and regulatory genes, the linolenic acid showed a dual effect on the expression of both classes of genes in *H. pylori*.

## Supplemental Information

10.7717/peerj.12270/supp-1 Supplemental Information 1 Gene expression raw data, IL-8 quantification, motility, adherence levels and biofilm formation

## Additional Information and Declarations

### Data Availability

The following information was supplied regarding data availability: The raw numeric data of RT-qPCR, IL-8 quantification, motility, adherence levels and biofilm formation are available in the Supplemental Files.
